# High-Performance Blue Quantum Dot Light Emitting Diode via Solvent Optimization Strategy for ZnO Nanoparticles

**DOI:** 10.3390/nano11040959

**Published:** 2021-04-09

**Authors:** Ji Xu, Lixi Wang, Xueliang Zhao, Yutong Shi, Yongjiao Shi, Ting Liu

**Affiliations:** 1 School of Electronic and Information Engineering, Nanjing University of Information Science & Technology, Nanjing 210044, China; 20201249400@nuist.edu.cn; 2 Joint International Research Laboratory of Information Display and Visualization, School of Electronic Science and Engineering, Southeast University, Nanjing 210096, China; 220181319@seu.edu.cn (Y.S.); 220181407@seu.edu.cn (Y.S.); 3 Department of Physics, College of Science, Beijing University of Chemical Technology, 15 East Road, Beijing 100029, China

**Keywords:** quantum dots (QDs), ZnO nanoparticles, solvent optimization, quantum dot light-emitting diodes (QLEDs)

## Abstract

Here, we report on the high-performance blue quantum dots (QDs) light-emitting diodes (QLEDs), in which the ZnO nanoparticles (NPs) are employed as the electron transport layer (ETL) and optimized with different alcohol solvents. The experimental results demonstrate that the properties of solvent used for ZnO NPs—such as polarity, viscosity and boiling point—play a crucial role in the quality of film where they modulate the electron injection across the QDs/ETL interface. The maximum current efficiency of 3.02 cd/A and external quantum efficiency (EQE) of 3.3% are achieved for blue QLEDs with ZnO NPs dispersed in butanol, exhibiting obvious enhancement compared with the other solvents. This work provides a new method to select proper solvent for ETL which can further improve the device performance.

## 1. Introduction

Quantum dots (QDs) have attracted much attention in the past few years because they possess unique properties, such as bandgap tunability, high photoluminescent quantum yield (QY), narrow full width at half maxima (FWHM), low-cost solution processing, and compatibility with flexible substrates [1,2,3,4,5,6,7]. Owing to these features, QDs have been extensively investigated for use in optoelectronic devices, such as light-emitting diodes (LEDs) [8], lasers [9], photodetectors [10], and solar cells [11]. In these applications, QD LEDs (QLEDs) have been of great importance in their application in display and lighting technologies. It has been over two decades since the first QLEDs were reported [12]. Researchers have made huge efforts to enhance the performance of QLEDs to be comparable with those of organic LEDs (OLEDs), which pushes QLEDs towards a candidate for single-material and full-color light sources [13,14,15].

However, there are still several problems in restricting the development of QLEDs [16,17]. For instance, the injection efficiency of carriers from the adjacent charge transport layers (CTL) into QDs is vital to the device performance considering the electron and hole need to be injected into the QDs layer successfully and form an exciton, followed by radiative recombination. To promote carrier injection/transport efficiency, various strategies have been devoted to material synthesis and device structure design [18,19]. Meanwhile, the fabrication process is complicated by processes such as modifying the structure of QDs or inserting a buffer layer composed of organic material, which leads to an increase in the production cost [16].

Taking the limiting factors mentioned above into account, it is necessary to exploit a facile way to promote carrier transport in order to obtain high-performance blue QLEDs. One promising approach is to modify the QDs/CTL interface [20]. The most commonly used electron transport layer (ETL) in the QLED is ZnO nanoparticles (NPs), which not only promote electron transport but also protect the QDs from moisture and oxygen [21]. The solvent selected to dissolve ZnO NPs influences the film quality during their spin coating on the QDs layer. Various solvents are used to disperse the ZnO NPs—including ethanol [21], butanol [22], and 2-methoxyethanol [23]—though there is a lack of systematic research on the effect of different solvent type for QLEDs. Therefore, we investigate the influence of solvents for ZnO NPs on the dynamic light scattering and morphology of ETL, as well as the optoelectronic properties of QLEDs. Our results indicate that QLEDs with butanol used for ZnO NPs achieve the highest performance because they have the best dispersion property and film quality, leading to the maximum current efficiency of 3.02 cd/A and external quantum efficiency (EQE) of 3.3%. Our work provides a simple but reliable method of improving the blue QLEDs performance.

## 2. Materials and Methods

### 2.1. Chemicals

Oleic acid (OA) (90%), trioctylphosphine (TOP) (90%), octadecene (ODE) (90%), CdO (99.5%), Zn acetate (99.99%), S powder (99.998%), Tetramethylammonium hydroxide (TMAH) (≥97%), poly(ethylenedi-oxythiophene):polystyrene sulphonate (PEDOT:PSS, AI 4083), and poly-*N*-vinylcarbazole (PVK) were obtained from Sigma-Aldrich.

### 2.2. Synthesis of Blue QDs

Blue-emitting CdZnS/ZnS QDs were synthesized according to the synthetic protocol reported previously with some modification [1] to the amount of sulfur in the core synthesis to adjust the emission wavelength. The resulting QDs were dispersed in octane with a concentration of 12 mg/mL.

### 2.3. Synthesis of Blue ZnO NPs

The ZnO NPs were synthesized as follows: first, a solution of 0.5 M tetramethylammonium hydroxide (TMAH) in ethanol was added to 0.1 M zinc acetate in dimethyl sulphoxide (DMSO). Then, the solvent was stirred in ambient conditions for about 1 h. After washing with acetone, the samples were dispersed with a concentration of 30 mg/mL in different solvents, including ethanol, butanol, methanol, and 2-methoxyethanol for comparison.

### 2.4. Fabrication of QLEDs

Patterned indium tin oxide (ITO) glass was chosen and cleaned as a substrate. After treatment under UV-ozone for 30 min, the 35 nm-thick PEDOT:PSS formed as a hole injection layer (HIL) was spin-coated (2500 rpm, 60 s) and baked at 140 °C for 30 min in the ambient atmosphere. Then, the coated substrates were transferred to the glove box. On top of HIL, the PVK (10 mg/mL in chlorobenzene) was spin-coated at 3000 rpm for 40 s. After annealing, the QDs (12 mg/mL in octane) layer was spin-coated as emitting layer (EML) (1500 rpm for 30 s) with post-baking at 150 °C. Subsequently, the ZnO NPs were spin-coated at 3000 rpm for 30 s. Finally, a 4 mm^2^ aluminum pad was deposited on the top of the device as an electrode.

### 2.5. Characterization

The absorption and photoluminance spectra were obtained by the UV-visible instrument and Edinburgh instruments’ FLS980 spectrometer, respectively. The film morphology was analyzed by the scanning electron microscope (SEM, JEOL-7800F) and atomic force microscopy (AFM, Veeco Dimension 3100). The size distribution of the NPs dispersing in solvents was determined by dynamic light scattering (DLS). Transmission electron microscopic (TEM) measurement was performed using a Tecnai G20 operating at 200 kV to obtain particle morphology of QDs. The electroluminance performance of QLEDs was measured using a Minolta luminance meter (LS-100) under ambient conditions.

## 3. Results

**Figure 1:**
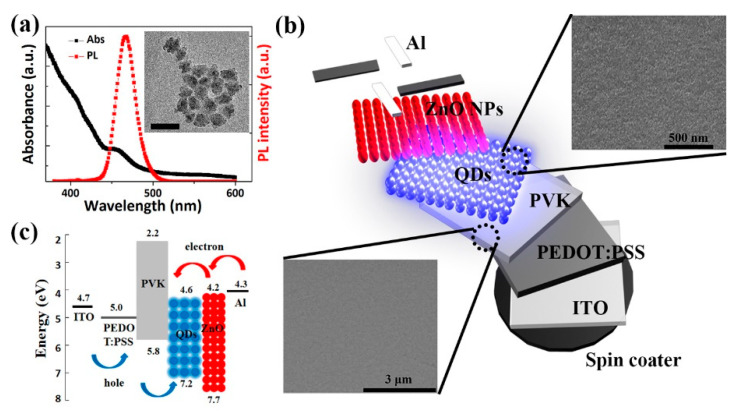
(**a**) Absorption and PL spectra of QDs. Inset is the TEM image of the synthesized QDs. Scale bar is 20 nm; (**b**) fabrication process of multilayered QLEDs. Insets are the SEM images of QDs layer (top) and PVK (bottom); (**c**) Energy level diagram for the various layers.

The PL and absorption spectra of the blue QDs in octane are shown in Figure 1a. The PL peak of QDs is located at 467 nm with an FWHM of 27 nm. The exciton absorption peak is 455 nm. The inset presents the TEM image of the QDs, indicating that the diameter of the QD is about 12 nm. Figure 1b displays the structure of the fabricated QLED—which consists of the layers ITO, PEDOT:PSS, PVK, QDs, ZnO NPs, and Al—in which the layers act as the anode HIL, HTL, EML, ETL, and the cathode, respectively. The inset shows the surface morphologies of compactly packed PVK and QD layers are relatively uniform, which is crucial to improving the overall performance of the device. As is shown in Figure 1c, PVK is used as the HTL because of the relatively deeper HOMO level compared with the other commonly used materials [24]. It is reported that the HOMO level has a greater influence on the hole transport than the hole mobility of the material [25]. As for the electron injection and transport, the ZnO NP with an electron affinity of 4.2 eV and an ionization potential of 7.7 eV is utilized as the ETL [26]. It can sufficiently improve the electron injection from the Al cathode into QDs. Moreover, because of the valence band offset at the QD/ZnO NPs interface, the NPs can also confine holes within the QD layer, which improves the charge recombination efficiency.

**Figure 2:**
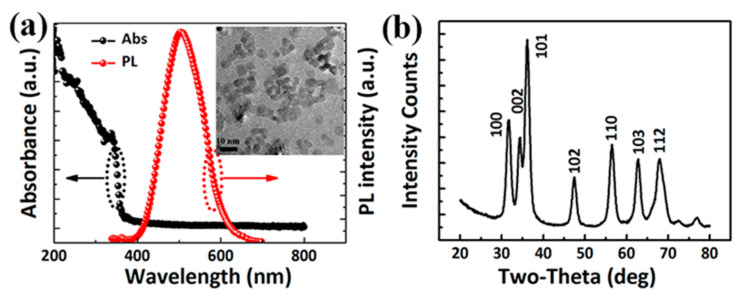
(**a**) Absorption and PL spectra of the ZnO NPs. Inset is the TEM image of the synthesized ZnO NPs; (**b**) XRD spectra of the ZnO NPs.

Figure 2a shows the absorption and PL spectra of the ZnO NPs, exhibiting a continuous absorption band with an edge at 355 nm. The bandgap Eg of ZnO NPs is calculated as 3.5 eV according to the absorption spectra. The relatively broad and visible PL emission of ZnO NPs reflects a recombination of electrons and holes, likely due to the traps or structural defects on the surface of the NPs [27]. The TEM image of the ZnO NPs indicates that the diameter of ZnO NPs is about 4 nm (Inset of Figure 2a). From the XRD pattern of the ZnO NPs (Figure 2b), our synthesized ZnO NPs show a typical wurtzite structure, which is in accordance with the formal literature (JCPDS card no.79-0207).

**Figure 3:**
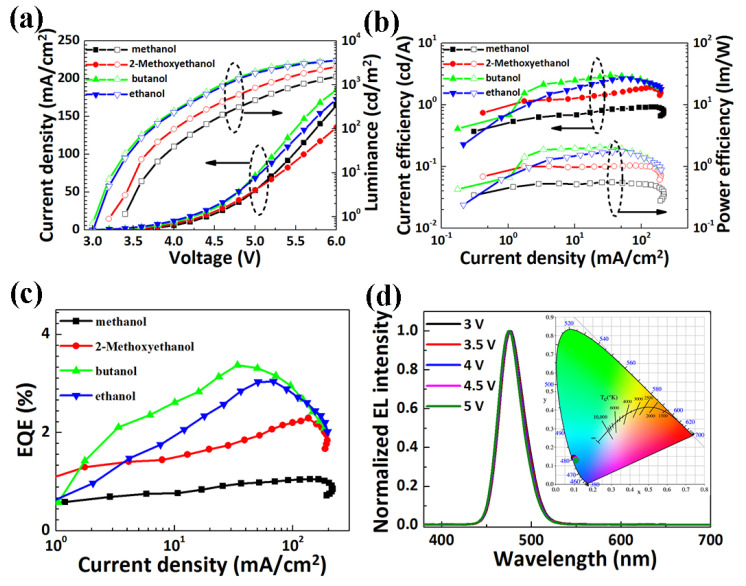
(**a**) Current density and luminance-voltage characteristics of the QLED with ZnO NPs dispersed in different solvents; (**b**) Current efficiency and power efficiency, and (**c**) EQE of QLED device with ZnO NPs dispersed in different solvents; (**d**) Normalized EL spectra of QLEDs with butanol. Inset shows the CIE coordinate of the device under different driving voltage.

Subsequently, we characterized the electroluminescent performance of blue QLEDs with ZnO NPs dispersed in different solvents. Figure 3a displays that the current density of QLED with butanol is larger than those with other solvents, indicating an enhancement of electron transport. It is noted that the luminance significantly increases in accordance with the increasing driving current. The luminance of the device with butanol at 5 V reaches 1992 cd m^−2^, which is the highest compared to the devices with other solvents at 5 V. Furthermore, the turn-on voltage is defined as the voltage corresponding to 1 cd m^−2^. In this case, the turn-on voltage for the device with methanol and butanol is 3.6 V and 3.1 V, respectively. The turn-on voltage of the ethanol device is similar to that of the butanol device due to the similar electron transport efficiency. On the other hand, the turn-on voltage of the device decreases from 3.6 to 3.1 V by employing ZnO dispersed in butanol, which may be caused by the improved electron transport efficiency. It can also be inferred that the properties of the solvents may play a primary role in improving the interface quality at the QDs/ZnO NPs interface. A detailed explanation will be discussed in the following section. The device with butanol shows that the maximum current efficiencies and power efficiencies of 3.02 cd/A and 2.06 lm/W, respectively (Figure 3b). That is a significant increase compared to that of the device with the other solvents. Meanwhile, simultaneous improvement in EQE has been achieved (Figure 3c). The maximum EQE of the device with butanol is 3.3%, much higher than that of the device with 2-methoxyethanol (2.3%), methanol (1.1%), and ethanol (3%). The enhancement of the device performance results from the improved electron transport due to the optimization of the QDs/ZnO NPs interface. The performance parameters of these devices are summarized in Table 1. Figure 3d shows the normalized EL spectra of QLEDs with ZnO NPs dispersed in butanol under different driving voltage. It is noted that no obvious change occurs for the EL spectra of devices under different driving voltage, indicating the exciton recombination zone is a preserved constant under different voltage. The inset of Figure 3d displays the CIE coordinate of the device under different voltage, thus verifying the stable emission of the device. Furthermore, the peak of EL is redshifted by 8 nm compared to that of the PL peak of the QDs, which may result from a combination of finite dot-to-dot interactions in the close-packed solid films and the electric-field-induced Stark effect [21].

**Figure 4:**
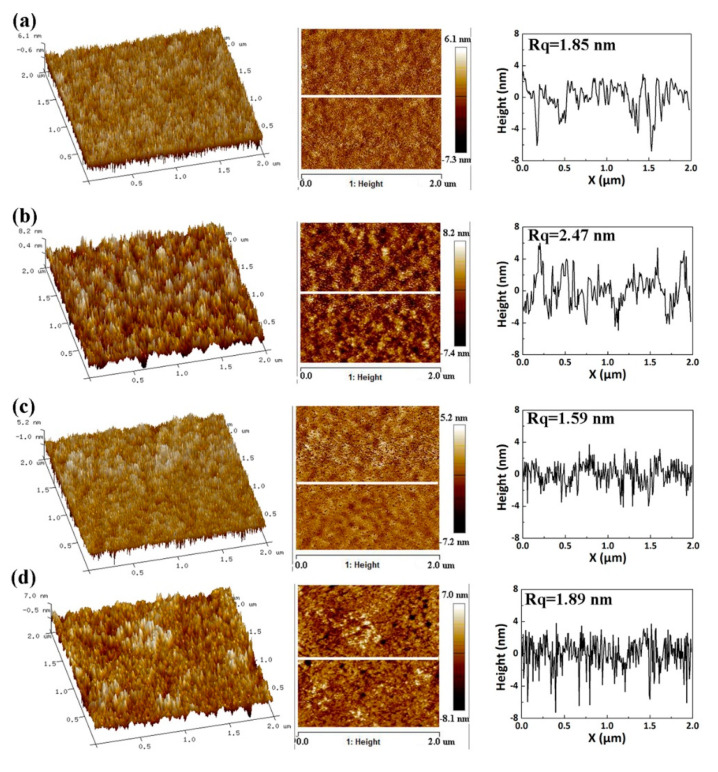
AFM characterizations of ZnO NPs on the blue QDs film with the solvent of (**a**) 2-Methoxyethanol, (**b**) methanol, (**c**) butanol, and (**d**) ethanol. For each sample, the pseudo-three-dimensional image, the height image, and the line-scan profile are shown from left to right.

AFM measurements of ZnO with different solvents deposited on the blue QDs were performed, showing the quality of the QDs/ZnO interface. The root mean square roughness (Rq) of ZnO NPs coated in different solvents varies between 1.59 and 2.47 nm, among which the ZnO NPs layer formed from butanol has the lowest Rq of 1.59 nm (Figure 4). Meanwhile, the line-scan profile of ZnO also confirms the improved quality of the QDs/ZnO interface when using butanol to disperse ZnO. It can be deduced that the QLED devices based on the ZnO NPs dispersed in butanol perform best, considering the relationship between the film roughness and carrier transport. The solvent property parameters are listed in Table 2 [28,29]. The properties of the solvent, such as polarity, viscosity, and boiling point, are different, leading to an obvious difference in the ZnO morphology, as shown in Figure 4. It is speculated that the higher viscosity and boiling point, along with lower polarity, are beneficial in forming a uniform and high-quality film of ZnO NP [30,31]. In addition, we suppose that the possible mechanism for the worse performance of 2-methoxyethanol may attribute to two reasons. On the one hand, the 2-methoxyethanol may damage the underlying QDs layers because of the high boiling point of the residual solvent. In addition, the viscosity of 2-methoxyethanol is greater than ethanol, which hinders the NPs from spreading out easily when dispersing. Such agglomeration has detrimental effects on the surface morphologies of ZnO ETLs [7]. However, the difference of surface morphologies may not be distinguished by AFM if the protruding structures appear in individual locations considering that the roughness result of AFM reflects the average value of film roughness in a certain area.

**Figure 5:**
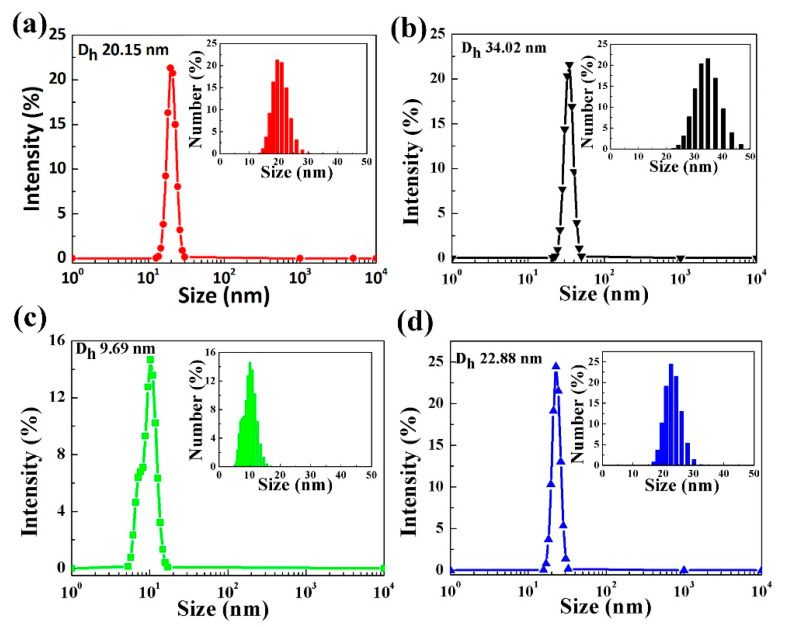
Size distribution of ZnO NPs determined in different solvents by DLS with (**a**) 2-Methoxyethanol, (**b**) methanol, (**c**) butanol, and (**d**) ethanol.

In order to further study the relationship between the solvent types and film roughness of ZnO NPs, dynamic light scattering (DLS) was measured for the ZnO NPs in different solvents as displayed in Figure 5. The size distribution of the ZnO NPs dispersed in solvents can be clearly seen. The hydrodynamic diameter (Dh) of ZnO NPs is approximately 9.69 nm in butanol, which is the smallest among these solvents. The smaller hydrodynamic diameter means less aggregation of the ZnO NPs due to their good dispersion in butanol. Different from the QDs with a large number of surface ligands, ZnO NPs are easy to aggregate in the solvent due to their lack of surface ligands [2]. The data in Table 2 also imply that butanol with the smallest solvent polarity of 3.9 is suitable for dispersing ZnO NPs and forming a stable solution for ETL. We can conclude that the employment of butanol as the solvent for ZnO NPs can promote carrier transport, due to the alleviation of the aggregation of ZnO NPs and improvement of ZnO NPs film quality.

## 4. Conclusions

In conclusion, we can report on an efficient method of solvent optimization to improve the performance of blue QLEDs. The relationship between the solvent property of ZnO NPs and the performance of the device can be analyzed by the morphology and dispersion studies of ZnO NPs, along with the optoelectronic characterization of QLED devices. The employment of butanol to disperse ZnO NPs can alleviate the aggregation of ZnO NPs, improve the ZnO NPs film quality, and promote electron transport. As a result, the maximum current efficiency of 3.02 cd/A and EQE of 3.3% are obtained based on the ZnO NPs dispersed in butanol, an enhancement factor of 10% compared with those of the device based on the other solvents. We believe that this method can lead to high-quality, stable, and low-cost electroluminescent devices based on QDs applications.

## Figures and Tables

**Table 1:** Summary of turn-on voltage, driving voltage, EQE, current efficiency, and power efficiency data of the QLED Device based on ZnO NPs dispersed in different solvents.

Type of Solvent	V_T_^a^ (V)	V_D_^b^ (V)	η_EQE_ (%)		η_A_ (cd/A)		η_P_ (lm/W)	
			Peak	@ 10^3^ cd m^−2^	Peak	@ 10^3^ cd m^−2^	Peak	@ 10^3^ cd m^−2^
2-Methoxyethanol	3.5	4.1	2.3	1.5	1.99	1.25	1.03	0.97
methanol	3.6	4.3	1.1	0.8	0.92	0.7	0.55	0.52
butanol	3.1	3.7	3.3	2.2	3.02	2.2	2.06	1.88
ethanol	3.2	3.7	3	1.6	2.67	1.6	1.75	1.35

^a^ Measured voltage when luminance was 1 cd m^−2^. ^b^ Measured voltage when luminance was 100 cd m^−2^.

**Table 2:** Summarized properties of different solvents.

	Polarity	Viscosity (mPa·s, 20 °C)	Boling Point (°C)
2-methoxyethanol	5.5	1.72	124
methanol	6.6	0.6	65
butanol	3.9	2.95	117
ethanol	4.3	1.2	79
